# Machine Learning Techniques for Soybean Charcoal Rot Disease Prediction

**DOI:** 10.3389/fpls.2020.590529

**Published:** 2020-12-14

**Authors:** Elham Khalili, Samaneh Kouchaki, Shahin Ramazi, Faezeh Ghanati

**Affiliations:** ^1^Department of Plant Science, Faculty of Science, Tarbiat Modarres University, Tehran, Iran; ^2^Faculty of Engineering and Physical Sciences, Centre for Vision, Speech, and Signal Processing, University of Surrey, Guildford, United Kingdom; ^3^Department of Biophysics, Faculty of Biological Science, Tarbiat Modares University, Tehran, Iran

**Keywords:** charcoal rot, gradient tree boosting algorithm, *Macrophomina phaseolina* (Tassi) Goid, machine learning, prediction

## Abstract

Early prediction of pathogen infestation is a key factor to reduce the disease spread in plants. *Macrophomina phaseolina* (Tassi) Goid, as one of the main causes of charcoal rot disease, suppresses the plant productivity significantly. Charcoal rot disease is one of the most severe threats to soybean productivity. Prediction of this disease in soybeans is very tedious and non-practical using traditional approaches. Machine learning (ML) techniques have recently gained substantial traction across numerous domains. ML methods can be applied to detect plant diseases, prior to the full appearance of symptoms. In this paper, several ML techniques were developed and examined for prediction of charcoal rot disease in soybean for a cohort of 2,000 healthy and infected plants. A hybrid set of physiological and morphological features were suggested as inputs to the ML models. All developed ML models were performed better than 90% in terms of accuracy. Gradient Tree Boosting (GBT) was the best performing classifier which obtained 96.25% and 97.33% in terms of sensitivity and specificity. Our findings supported the applicability of ML especially GBT for charcoal rot disease prediction in a real environment. Moreover, our analysis demonstrated the importance of including physiological featured in the learning. The collected dataset and source code can be found in https://github.com/Elham-khalili/Soybean-Charcoal-Rot-Disease-Prediction-Dataset-code.

## Introduction

The production of global crops has to be doubled by 2050 to meet the increasing needs of the world’s population (Khalili et al., 2019). Plant diseases are the lead causes of extensive economic losses in the agricultural industry around the world. Recent statistics have confirmed that there is a decline of worldwide crop yields by 14% worldwide due to plant diseases, weeds and insects, and hence, early detection of diseases is of a key importance to prevent disease spread and reduce damage to crop production (Martinelli et al., 2015). *Macrophomina phaseolina* (Tassi) Goid causes rot diseases in about 700 plant species. It is an extremely robust soil-borne fungus that damages several crops i.e., cotton, grains, oilseeds, legumes, jute along with fruits and vegetable plants (Ambrosio et al., 2015; Sun et al., 2016). A wide range of physiological, morphological, and pathogenic diversity enables *M. phaseolina* to adapt across various climatic conditions (Ambrosio et al., 2015). Moreover, sclerotia and chlamydospores structures enable the fungus to survive in the soil for a longer period (Katan, 2017). Gaige et al. (2010) described that the disease is dispersed by infected plant residues, wind, and soil. The infestation of *M. phaseolina* pathogen may occur at any growth stage whereas symptoms often appear after the midseason or at maturity i.e., growth stage R7 where yellowing of the leaves and yellow pods can be observed (Hartman et al., 2016). Other symptoms may include the development of “blackleg” in infected plants which results in weaker plants and lower productivity (Santos et al., 2016). The infected plants ultimately die due to various reasons such as vascular blockages that weaken the nutrient transport (Santos et al., 2016) or exposure to phytotoxic metabolites released by *M. phaseolina*.

For decades, agricultural management strategies for controlling plant diseases were mainly based on cultural practices e.g., soil solarization, crop rotation, cultivation of tolerant cultivars, alone or combined with other techniques such as low doses of pesticides and biological agents (Holmes et al., 2020). Generally, fumigants and fungicides are used to control *M. phaseolina* infections in crops that can be ineffective and inefficient due to different environmental factors as reported in Abbas et al. (2019). In a work presented by Khalili et al. (2019), a higher dose of fungicides was suggested for an economical yield. An increased dose of these chemicals leads to concerns over the long-lasting harmful impacts of pesticides on human health and ecology as agricultural run-offs contain pesticides which pollute the water resources (Chamorro et al., 2015; Pastrana et al., 2016). Moreover, the bioaccumulation of these toxic compounds in the food chain and further ingestion by bird populations and mammals pose vital health-associated threats (Brevik et al., 2020).

The efficient detection of diseases can be a key factor in the sustainability of the agroecosystem. The developments in molecular biology and biotechnology have improved the detection of plant diseases. Reverse Transcription Polymerase Chain Reaction (RT-PCR), Enzyme-Linked Immuno-Sorbent Assay (ELISA), and Western blotting (WB) are examples of plant disease diagnostic techniques (Jeong et al., 2014; Golhani et al., 2018). However, these techniques are not able to predict the fungal disease despite their diagnostic efficiency (Sakudo et al., 2006; Thanarajoo et al., 2014). Moreover, RT-PCR, ELISA, and WB are limited in terms of cost-effectiveness, efficiency, and accuracy for the prediction of disease infestation (Eun et al., 2002).

Therefore, an automated diagnostic system is important to prevent and control diseases in soybean. It would minimize the yield and economic losses, reduce pesticide residues, and enhance product quality (Nagasubramanian et al., 2018). Effective soybean disease classification is critical to predict the disease at the early stages. Machine learning (ML) techniques have found application in several areas of research such as crop management, yield prediction (Chlingaryan et al., 2018), disease detection (Kouchaki et al., 2019), and weed detection crop quality (Liakos et al., 2018; Wang et al., 2019). These algorithms learn through examples (training data), to predict the unseen data (Ashfaq et al., 2017). Researchers have also applied learning algorithms in predicting the pest attack and disease infestation in crops (Patricio and Rieder, 2018).

In this work, a number of ML algorithms, including linear regression with L1 and L2 regularization terms (LR-L1 and LR-L2), neural network (Multilayer perceptron, MLP), random forest (RF), gradient tree boosting (GBT), and support vector machines (SVM) were developed and compared for soybean disease prediction. These algorithms have been used to classify healthy and infected plants using spectral imaging data of aerial parts of plants (Ur Rahman et al., 2017). ML methods have also been proven successful in monitoring morphological traits (Singh et al., 2017; Mochida et al., 2019). Nonetheless, variations in symptoms may lead to an improper prediction due to dynamic nature of plant changes. Consequently, the appearance-based identification of diseases is not reliable enough to accurately detect unhealthy plants especially in the early growth stages. An appropriate method is vital for detection of the causal agent as charcoal rot does not have any visible symptoms until the midseason (Sladojevic et al., 2016). Hence, we have proposed a hybrid feature set for the prediction of charcoal rot disease using physiological features and morphological characteristics (including growth attributes as well as yield-related features). As a result, ML algorithms are trained and assessed based on the hybrid feature sets of healthy and infected soybean plants. The available dataset contains both experimental setups and real cultivation conditions in the field. The work shows the application of ML techniques to detect unhealthy plants from the healthy group.

Currently, no public dataset for soybean charcoal rot disease classification is available. The applicability and success of supervised ML algorithms on predictive disease modeling have been reported but for other diseases and mainly based on image datasets. Therefore, our main focus is to suggest a set of informative features to enhance charcoal rot disease prediction as well as providing a comprehensive comparison of several ML techniques.

## Materials and Methods

### Dataset Collection

Soybean (*Glycine max* L.) plants were collected from 10 different areas of Mazandaran province which is the most prolific geographical region for the production of soybean in Iran (Supplementary Figure 1). Soybean healthy plants were collected based on the symptomless features of leaf, stem, and root of mature during the ripeness stage. In this study, the R7 was chosen for infected plants based on the physical properties e.g., the existence of bright gray and sclerotia on the stem and root and suspicious of diseases. All samples were transferred to the laboratory of the Agricultural and Resource Research Center of Mazandaran (Iran) and stored at 4°C until further analysis. Overall, 2,500 plants were randomly chosen from healthy and infected plants.

### Symptoms of Infected Soybean Samples

The infection of this pathogen is observed on all parts of the plant i.e., branches, leaves, pods, petioles, root, stem, and seeds on soybean (Gupta et al., 2012), however, the key indications of disease are observed after the flowering stage in infected plants, i.e., R7 stage, especially in low humidity level and high-temperature conditions (Schoving et al., 2020). Chlorosis of leaves, premature defoliation, and reduced vigor are the major symptoms observed in the infected plants (Romero Luna et al., 2017), which result in reduced productivity, sterility of pod, and formation of crinkled and tiny seeds. A brown discoloration in the vascular tissues of the taproot advanced into the stem is seen in infected plants. An appearance of powdery black sclerotia is found under the epidermis and root at the seed formation stage in the infected plants. Sometimes, the plant symptoms of this disease are confused with other plant abiotic stresses like drought or abiotic stress like cyst nematode, therefore the detection of this disease based on morphological aerial plant parts is challenging (Sanchez et al., 2019).

### Laboratory Assessment

#### Determination of Morphological Parameters

All soybean healthy and infected samples were collected and transferred to the laboratory and then cleaned with tap water until all noticeable soil and sand spots were removed. Forceps were used to remove the remaining particles manually. The mature seedlings were observed on the 54th day after sowing while mature pods were observed on the 80th day after sowing. Specifically, the length and thickness of the stem and root as well as length, width, and thickness of the seeds were examined (Fenta et al., 2014). Length of the mature seedlings, stem and root of a soybean plant was measured and reported in cm. A pair of calipers were used to measure the length of root, pods and seeds and thickness of the seeds. Meanwhile, the thickness of the seeds was measured using a micrometer screw gauge. At each harvest, the number of seeds and pods per each plant were manually categorized based on the date of pod or flowering set to count the numbers of empty and filled pods (with or without rudimentary seeds) (Joshi et al., 2015).

#### Determination of Fresh Weight and Dry Weight

An electronic top pan balance was employed to calculate the fresh weights (FW) of soybean seedlings, stems and roots (Model BL-210-S, Sartorius, Germany). On the other hand, Samples were oven-dried at 70 ± 2°C for 72 h for weighting the dry weight (DW). DW and FW were stated in grams per plant (Schnyder and Baum, 1992).

### Yield-Related Parameters Assessment

#### Seed Quality Index

Germination percentage (GP) and Seedling vigor index (VI) of the soybean seeds were measured after 2 weeks. Daily observations of seed emergence were carried out. Seed germination percentage is calculated as follows (Islam et al., 2009):

(1) $$\mathrm{GP}=\,\frac{\sum \left(N-i\right)\,\times \text{Gi}}{\text{N}\times \text{GN}}\times \,100$$

where i and N are the number of days since the day of sowing and the total number of days, respectively. Gi and GN are the number of seeds germinated on day i, and the total number of germinated seeds, respectively.

Furthermore, seedling vigor index (SI) is calculated by Islam et al. (2009):

(2) VI=GP×SL×100

where, GP is germination percentage, SL is the seedling length in cm.

#### Thousand Seed Weight

It is highly useful for calculating the optimal seeding rate for a given crop type. A large variation was observed across measured seed weights. We grouped the weights in two groups of light (<100 g) and medium or intermediate (>100–200 g). Individual seeds were weighted by calculating the weight of 1,000 fresh seeds (empty seeds were discarded). The frequency distribution of the seed weight was determined by checking its normality using the K-S test (Brzezinski et al., 2015).

### Determination of Physiological Parameters

After the harvest, the soybean seeds were taken to the laboratory for physiological quality assessments, through the following tests:

#### Protein Content and Seed Oil Content

The oil content was calculated and expressed in percentage of the dry matter using petroleum ether and in a Soxhlet instrument (technique 920.85, AOAC, 1990). The protein content was obtained and expressed in percentage of the dry matter by indicating the total nitrogen based on the micro Kjeldahl method (technique 920.87, AOAC, 1990) by considering a 6.25 conversion factor (Vaknin et al., 2011).

#### Amount of Chlorophyll and Carotenoid

Samples with 0.1 g of leaves (fresh material) was chosen randomly. Each soybean sample was grounded in 0.5% (w v^–1^) magnesium carbonate and 10 mL of 80% acetone. Then, 10 ml of 100% acetone was added. A spectrophotometer was used to measure the absorbance (Jenway 6105 UV/VIS) in 663 nm (chlorophyll *a*—Chl *a*), 645 nm (chlorophyll *b*—Chl *b*) and 480 nm (carotenoids—C_*x*__+__*c*_) wavelengths. Equations described by Hendry and Price (1993) was employed to calculate the chlorophyll concentrations. The fraction of photosynthetically active irradiance absorbed by the leaf (α) depends on the chlorophyll content (μmol m^–2^) and it was calculated as α = Chl_*to*__*t*_/(Chl_*to*__*t*_ + 76) by considering the work of Evans and Poorter (2001). The data obtained was subject to a regression analysis using the SigmaPlot 8.02 package for Windows.

### Morphological and Physiological Feature Extraction

An observation was conducted to check the morphological and physiological characteristics of each soybean plants. In order to carry out the ML experiments, two categories were considered; healthy and infected. Healthy (negative) and infected (positive) plants were separated based on symptoms of charcoal rot. Appropriate attributes were selected based on the differences between the healthy and infected plants. Some data within each category of healthy or infected samples had very similar feature values. Therefore, as a preprocessing step, we dropped all but one of very similar data samples as they would not add any extra information to the learning or validation of the proposed pipeline. Finally, 1,000 healthy soybean plants (negative) and 1,000 infected plants (positive) were selected for charcoal rot disease prediction (Supplementary Table 1).

### Feature Selection

Feature selection was designed and optimized to enhance the performance and generalizability of ML models. In order to select the relevant features, analysis of variance and *F*-test were used (Elssied et al., 2014). These analyses were based on *p*-value for feature selection by skipping the irrelevant attributes from the data set (Eskandari and Javidi, 2016). *F*-test was performed to compute the statistical significance value and to calculate the *p*-value for the difference in means at the 5% level of significance. We finally ended up with a list of 21 features to be analyzed by ML techniques. Results of the *F*-test confirmed that morphological and physiological characters parameters were among the most important features for prediction of charcoal rot disease in soybean (Table 1).

**TABLE 1:** List of features for prediction of charcoal rot disease in soybean.

Features		
Morphological		
Growth attributes	Yield- related	Physiological
Stem length	Germination percentage	Seed oil content
Root length	Seedling vigor index	Amount of chlorophyll
Thickness of seed	Thousand seed weight	Amount of carotenoid
Stem bark thickness	Number of pods per plant	Protein content
Root bark thickness	Number of seeds per plant	
Stem fresh weight	Empty pods per plant	
Stem dry weight		
Root fresh weight		
Root dry weight		
Seedling fresh weight		
Seedling dry weight		

### Computational Methods for Predicting Infected Soybeans

**FIGURE 1:**
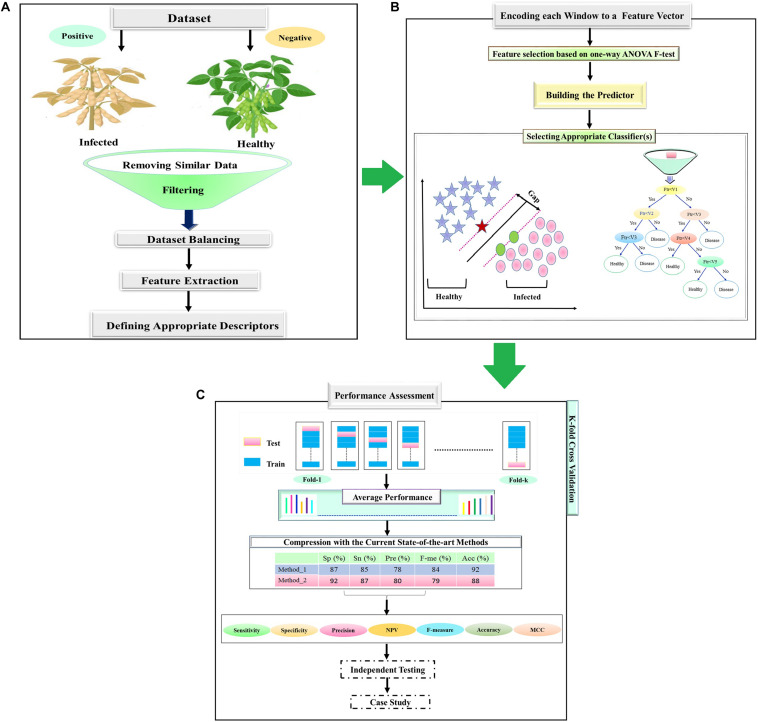
Flowchart of the statistical and ML techniques for the detection of charcoal rot disease. **(A)** Data set creation. **(B)** Feature selection and design. **(C)** ML models train and test.

Our pipeline for predicting infected soybean has four main steps: (1) data gathering; (2) feature extraction; (3) training the predictors; and (4) performance assessment. These steps have been described and have been schematically shown in Figure 1. Data gathering is the first step of the healthy soybean prediction (Figure 1A). After creating the positive and negative datasets, incomplete instances were removed. In order to have a balanced positive and negative dataset, a random subset of the negative dataset with an equal number of positive samples was selected. In the feature extraction step, the positive and negative samples (soybeans) are coded into numerical feature vectors to be used to learn the classifiers.

There is a variety of classifiers that can be learned and based on the performance of different classifiers, a suitable classifier can be selected (Figure 1B). A standard procedure for assessing the performance of a classifier is *k*-fold cross-validation. In this process, the available dataset is randomly divided into *k* subsets without an overlap. Then, *k*− 1 of them is utilized as a training dataset, and the remaining as the test set for evaluating the model (Lyons et al., 2018). This process is repeated *k* times to allow every subset to be used precisely once as the test set. Finally, the average performance for all *k* test sets is calculated (Figure 1C). The most important performance assessment measures are used in the prediction of the healthy soybeans are described in the following subsections. All of these measures are based on the four basic elements of the confusion matrix (true positive, false positive true negative, and false negative represented as TP, FP, TN, and FN, respectively).

### Machine Learning Methods

After the data collection and feature extraction steps, six ML techniques (LR-L1, LR-L2, MLP, RF, GBT, and SVM) were developed and applied to the training set. We used 10-fold cross-validation while the threshold was set based on the training data considering false positive and false negative rates. All the ML techniques were run by the open-source ML toolkit scikit-learn (version 0.20.1) in python 3.6.7. The parameters of the models (e.g., number of ensembles for RF or GBT) were optimized through an internal cross-validation on the training data. This was done by a grid search over a range of values and selecting parameters that generated the best area under the area under the receiver-operator characteristic (ROC) curve (AUC). The model with the highest performance was reported in the paper.

#### Regularized Logistic Regression (LR-L1 and LR-L2)

LR is a linear classification model that predicts binary outcomes based on a set of explanatory variables (i.e., features). This model is performed using LIBLINEAR library and L1 or L2 regularizations (LR-L1 and LR-L2). L1 regularization and L2 regularization are two common techniques to reduce the model over-fitting (Couronne et al., 2018).

#### Multilayer Perceptron (MLP)

MLP maps the input data to a non-linear latent representation. MLP contains several fully connected layers of nodes in which a non-linear activation function is considered for each node, except at the input layer. MLP employs back-propagation for training (Breiman, 2001) and has shown to be a highly applicable network, thus a popular choice among researchers (Shan et al., 2018). Two hidden layers of size 10 and 4 and Adam optimization were considered in this work.

#### Random Forest (RF)

RF is a non-linear ensemble method that consists of multiple decision trees. The final prediction is determined from the results of the individual trees (Basu et al., 2018), which improves the generalization ability of the model for a better prediction. The accuracy of an individual tree and a correlation between these trees are key points in the generalization ability of RF. RF is not usually sensitive in the choice of parameter selections (Teixeira et al., 2013).

#### Gradient Tree Boosting (GBT)

GBT (Friedman, 2002) is another ensemble algorithm based on decision trees that can be considered for both classification and regression problems (Cheng et al., 2018). In contrast to RF, this model sequentially builds decision trees by a weighting strategy to put more emphasis on harder samples. A weighted majority vote is then used to make the final prediction.

#### Support Vector Machines (SVM)

SVM aims to find a hyperplane that minimizes the structural risk (Czarnecki and Tabor, 2015) in kernel space. Gaussian radial basis function, Linear, and polynomial are several common kernel functions. SVM has two important hyperparameters, the kernel coefficient γ and the penalty parameter C. This model follows two goals of finding a low complexity model that best separates the data to have a better generalizability ability (Uddin et al., 2019). Linear kernel was considered in this work.

### Model Evaluation Criteria

The considered ML classification models are evaluated by calculating several evaluation parameters, true positive (TP) that indicates the number of correctly classified infected plants, true negatives (TN) that indicates the number of correctly classified healthy plants, false positives (FP) that denotes the number of healthy plants incorrectly classified as infected plants and false negatives (FN) that represents the number of infected plants incorrectly classified as healthy plants. The classification performance is often evaluated by accuracy, specificity, sensitivity, precision, Negative Predictive Value (NPV), F1 score and, Matthews Correlation Coefficient (MCC) value as shown in Figure 1C. Besides, we also assessed AUC as an indicator of model performance. The threshold for reporting the classification performance on the test sets was set on the train data. All performance criteria in this work are explained as follows:

#### Accuracy

Accuracy (Acc) is a ratio between the correctly classified data points to the total number of samples as described by Sokolova et al. (2006):

(3) $$\text{Acc}=\frac{T\,P+T\,N}{T\,P+F\,P+T\,N+F\,N}\times 100\%$$

#### Sensitivity and Specificity

Sensitivity describes the correctly classified positive samples to the total number of positive samples:

(4) $$\text{Sensitivity}=\frac{T\,P}{T\,P+F\,N}\times 100\%$$

whereas specificity is stated as a ratio of the correctly classified negative samples to the total number of negative samples:

(5) $$\text{Specificity}=\frac{T\,N}{T\,N+F\,P}\times 100\%$$

#### Precision

Precision or positive prediction value (PPV) shows the correctly classified positive samples to the total number of samples predicted as positive and described by Sokolova et al. (2006) as:

(6) $$\text{Precision}=\frac{T\,P}{T\,P+F\,P}\times 100\%$$

#### Negative Predictive Value (NPV)

Inverse precision, or true negative accuracy measures the proportion of negative samples that were correctly classified to the total number of negative predicted samples (Sokolova et al., 2006) as:

(7) $$\text{NPV}=\frac{T\,N}{F\,N+T\,N}\times 100\%$$

#### F-Measure

F-measure shows the harmonic mean of recall and precision and calculated as:

(8) $$\mathrm{F1score}=\frac{2\,T\,P}{2\,T\,P+F\,P+F\,N}\times 100\%$$

#### Matthews Correlation Coefficient (MCC)

MCC shows the correlation between true and predicted labels and described in Boughorbel et al. (2017) as:

(9) $$\text{MCC}=\frac{T\,P\times T\,N-F\,P\times F\,N}{\sqrt{\left(T\,P+T\,N\right)\,\left(T\,P+F\,N\right)\,\left(T\,N+F\,P\right)\,\left(T\,N+F\,N\right)}}\times 100\%$$

#### Area Under the ROC Curve (AUC)

ROC has been used over the past years within ML community to visualize and evaluate the trade-off between the true positive rates and the false-positive rates (Fawcett, 2006). In order to compare classifiers, ROC can be reduced to the single scalar value called the area under the curve (AUC) and defined as the area under the ROC curve, a measure of the quality of the classification (Marrocco et al., 2008). AUC is not impacted by the arbitrary selection of a specific classification threshold and we thus use it as the primary evaluation metric.

### t-Distributed Stochastic Neighbor Embedding (t-SNE) Data Visualization

The t-Distributed Stochastic Neighbor Embedding (t-SNE) has been successfully applied to visualization problems. Schubert and Gertz (2017), described that it attempts to preserve pairwise distance distribution of points in the lower dimensions. As the prediction in the lower dimensions includes the distribution of relative distances, it needs large data points to determine an expressive depiction. The t-SNE is a new technique in ML, which has been employed in biological data analysis (Grimes et al., 2013; Irish, 2014; Dimitriadis et al., 2018). It has also been successfully applied to visualize the infected rice leaf data in Zhang et al. (2020). In our work, t-SNE was used to visualize distinctions among positive (infected) and negative (healthy) samples.

## Results

### Model Verification and Evaluation

**FIGURE 2:**
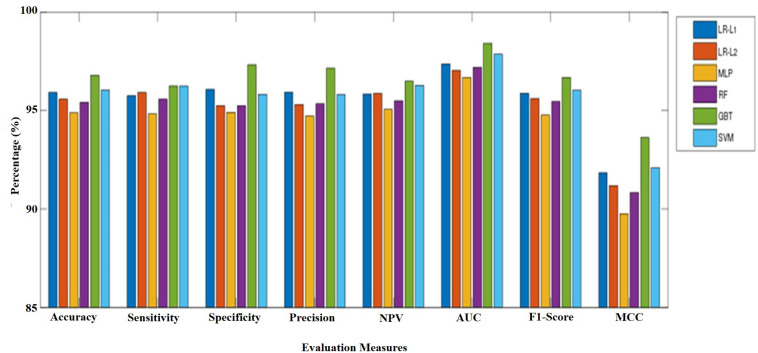
A comparison of different evaluation criteria for the prediction of healthy and infected soybean plants with charcoal rot disease considering different ML algorithms.

We employed 10-fold cross-validation to measure and relate the strength and trustworthiness of all models as a model build by only one random scale may tend to be over-fitting or occasional. The mean performance of six ML models for the test sets were shown in Table 2 and Figure 2. MLP performed the worst in terms of all the evaluation criteria with the lowest accuracy (94.88%), sensitivity (94.83%), specificity (94.92%), precision (94.72%), NPV (95.06%), F1 score (94.77%), and MCC (89.76%). The final analysis shows that GBT classifier performed the best with the highest classification accuracy (96.79%), specificity (97.33%), precision (97.16%), NPV(96.49%), F1 score (96.68%), and MCC (93.62%). SVM classifier is the second best with a classification accuracy of 96.04%, with TP (1150) and specificity (95.83%), precision (95.81%), NPV (96.29%), F1 score (96.03%), and MCC (92.09%) and LR-L1, LR-L2, and RF attained an average accuracy of more than 95%. Similarly, sensitivity for GBT and SVM were almost the same and ranked the highest. LR-L1 and LR-L2 also performed quite well with only slightly lower than GBT and SVM; their sensitivity was more than 95%. It could be summarized that the GBT and SVM models outperformed the other six models for the prediction of charcoal rot disease.

**TABLE 2:** Performance comparison of various ML techniques on the full features for prediction of soybean charcoal rot disease.

Method	TP	FP	TN	FN	Accuracy	Sensitivity	Specificity	Precision	NPV	F1 score	MCC	AUC
LR-L1	1153	47	1149	51	95.92 ± 8.32	95.75 ± 8.74	96.08 ± 7.93	96.01 ± 8.05	95.84 ± 8.60	95.88 ± 8.39	91.84 ± 16.63	97.37 ± 5.82
LR-L2	1143	57	1151	49	95.58 ± 9.00	95.92 ± 8.90	95.25 ± 9.16	95.31 ± 9.11	95.87 ± 8.90	95.61 ± 8.99	91.18 ± 17.99	97.05 ± 6.58
MLP	1139	61	1138	62	94.88 ± 9.58	94.83 ± 10.66	94.92 ± 8.51	94.72 ± 8.97	95.06 ± 10.16	94.77 ± 9.83	89.76 ± 19.14	96.69 ± 7.39
RF	1143	57	1147	53	95.42 ± 9.64	95.58 ± 9.27	95.25 ± 10.01	95.34 ± 9.80	95.50 ± 9.47	95.46 ± 9.54	90.83 ± 19.28	97.20 ± 6.35
**GBT**	**1168**	**32**	**1155**	**45**	**96.79 ± 6.49**	**96.25 ± 7.88**	**97.33 ± 5.16**	**97.16 ± 5.55**	**96.49 ± 7.29**	**96.68 ± 6.75**	**93.62 ± 12.90**	**98.42 ± 3.42**
SVM	1150	50	1155	45	96.04 ± 7.55	96.25 ± 7.88	95.83 ± 7.26	95.81 ± 7.36	96.29 ± 7.76	96.03 ± 7.61	92.09 ± 15.10	97.86 ± 4.65

### Determination of the Prediction Performances

ROC curve is one of the most robust approaches for evaluating ML techniques (Bradley, 1997). Here, the ROC curve was generated by varying the output threshold of the LR-L1, LR-L2, MLP, RF, GBT, and SVM classifiers and plotting the true positive rate (sensitivity) against the false positive rate (1—specificity) for each threshold value. An accurate classifier leads to a ROC curve which is close to the left-hand and top borders of the plot and hence AUC can be used as a performance measure (Robin et al., 2011). The maximum value of AUC is 1 while weak classifiers and random guessing have AUC values close to 0.5. We plotted the ROC curves and calculated the AUC for six models based on 10-fold cross-validation for prediction of charcoal rot disease. The evaluation was performed using 2,000 data which consists of 1,000 positive and 1,000 negative samples. In Supplementary Figure 2, the ROC curve of the GBT model is highlighted by the red color with the highest AUC value of 98%. Results demonstrated that the average AUC values of LR-L1, LR-L2, RF, and SVM were very close (97%), which means that the four models have equal sorting or accumulation ability in prediction probability. Meanwhile, MLP model gave the lowest AUC value (96%). GBT is a robust prediction system for charcoal rot disease on soybean considering AUC as the performance measure.

### GBT Model Performance

**FIGURE 3:**
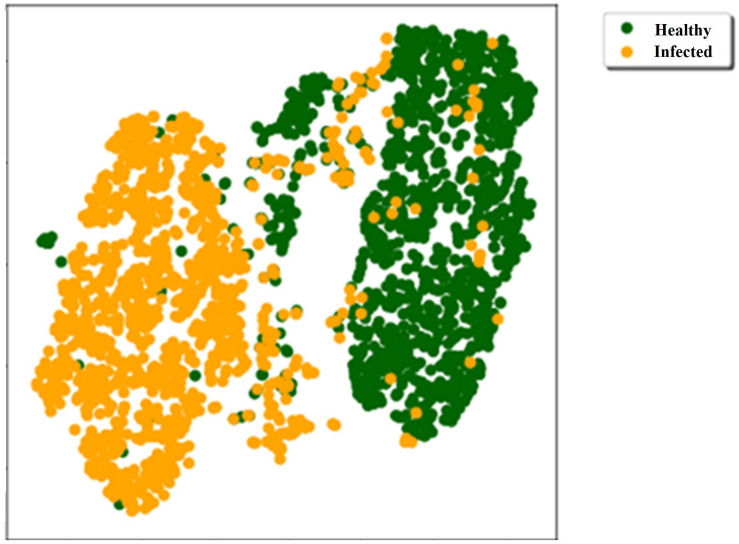
Two-dimensional t-SNE visualization of the data.

In the proposed system, we have classified healthy and infected plants of soybean dataset learning various ML classifiers on a hybrid feature set. After classification, we have calculated and compared their performance scores. The t-SNE was also applied to our dataset (Figure 3) to visualize the data in two-dimensions. As can be seen, most of the healthy and infected samples shape their clusters, although some characters of healthy and infected plants were identical which had the lowest difference in some physiological and morphological features. Consequently, having only 32 + 45 samples that were not correctly classified in our 10-fold cross-validation, demonstrates the application of ML techniques to classify most of such samples.

### Effectiveness Analysis of Feature Selection

To further evaluate the effectiveness of the full features on ML performance for prediction of charcoal rot, we took 12 morphological features for classification algorithms (LR-L1, LR-L2, MLP, RF, GBT, and SVM). Then, the prediction results are evaluated on the 12 features using the 10-fold cross-validation. For accuracy, GBT reached the highest value of 96.13%, followed by SVM and LR-L1 which performed only slightly lower than GBT with an average accuracy of 95.58%. The lowest classification accuracies of 94.50%, was resulted from MLP. The averaged prediction performance is listed in Table 3 and compared with that the full feature set. As can be seen from Table 2, the accuracy, sensitivity, specificity, precision, NPV, F1 score, MCC, and AUC of the full features are slightly higher to the morphological feature set. As shown in Tables 2, 3, the GBT algorithm has a higher performance by considering the hybrid feature set in comparison to the morphological features (96.79% vs. 96.13%).

**TABLE 3:** Performance comparison of various ML techniques based on 12 morphological features for prediction of soybean charcoal rot disease.

Method	Accuracy	Sensitivity	Specificity	Precision	NPV	F1 score	MCC	AUC
LR-L1	95.58 ± 8.51	95.25 ± 10.27	95.92 ± 6.97	95.66 ± 7.59	95.61 ± 9.28	95.41 ± 8.97	91.21 ± 16.92	97.24 ± 5.84
LR-L2	94.96 ± 9.63	94.50 ± 12.51	95.42 ± 7.32	94.99 ± 8.32	95.15 ± 10.55	94.64 ± 10.54	90.03 ± 18.99	96.96 ± 6.40
MLP	94.50 ± 8.64	94.17 ± 12.04	94.83 ± 5.88	94.46 ± 6.57	94.90 ± 10.32	94.17 ± 9.41	89.18 ± 16.98	97.29 ± 5.45
RF	95.46 ± 9.33	95.08 ± 10.33	95.83 ± 8.41	95.64 ± 8.88	95.31 ± 9.73	95.35 ± 9.62	90.93 ± 18.63	97.12 ± 6.35
**GBT**	**96.13 ± 7.64**	**95.92 ± 8.34**	**96.33 ± 6.97**	**96.22 ± 7.22**	**96.05 ± 8.04**	**96.06 ± 7.78**	**92.26 ± 15.27**	**98.00 ± 4.28**
SVM	95.58 ± 7.73	95.67 ± 9.05	95.50 ± 6.53	95.34 ± 6.92	95.88 ± 8.49	95.48 ± 7.99	91.20 ± 15.41	97.46 ± 5.51

### Feature Ranking

**FIGURE 4:**
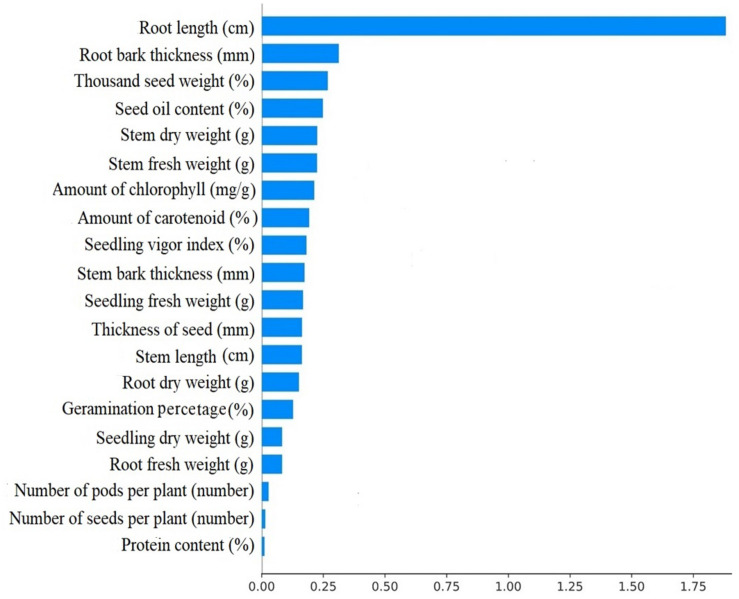
Mean SHAP values for the GBT model.

Table 4 shows the importance of the features by considering the ML models. The features were ranked according to their importance in the classification. The incremental usefulness is important in relevance from the perspective of feature ranking where the presence of such features enhances the performance of a classification system. The top 10 features ranked by each ML algorithm in this work are represented and highlighted by different colors in Table 4. To further understand the importance of individual features on model predictions, SHAP analysis (SHapley Additive exPlanations) was performed on the GBT model, and the results are presented in Figure 4. SHAP values can be used to interpret the impact on model prediction of the value of a given feature, in comparison to a baseline value (Padarian et al., 2020). According to the results, top features were mostly among the physiological features showing their importance in comparison with the morphological features for predicting the early stage of charcoal rot disease on soybean. Observing protein content, seed oil content and amount of chlorophyll in the top 10 feature means that they are predictive features for all the ML methods. The amount of amount of carotenoid and empty pods per plant is listed in the top 10 by all of the methods except MLP and GBT. Following thousand seed weight, thickness of seed, and number of seeds per plant are selected by at least four ML methods. Root length, stem bark thickness, root bark thickness, and seedling vigor index features are examples of the least informative features. On the other hand, features that are not in this list or are just selected by one method can be categorized as the least informative features. This information is significant as the most important features can be checked first to evaluate the seeds.

**TABLE 4:** Feature ranking results for various ML techniques.

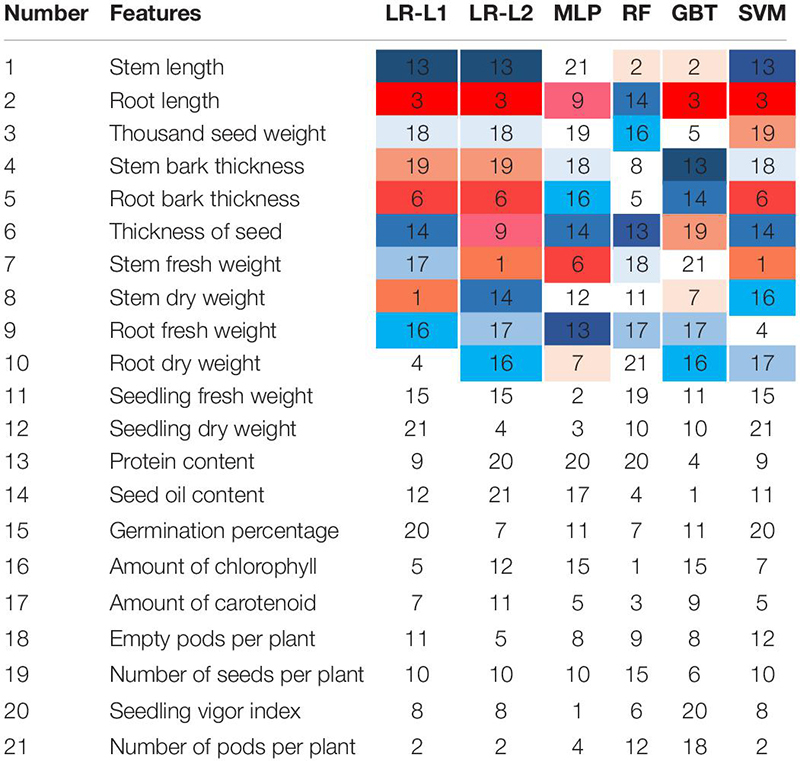

**Colors indicate the top 10 features ranked from high (blue) to low (red).**

## Discussion

Fungal diseases can be predicted through direct or indirect procedures. Direct procedures include polymerase chain reaction, immunofluorescence, fluorescence *in situ* hybridization, ELISA, flow cytometry, gas chromatography-mass spectrometry, and Western blotting. These could be used for high-throughput analysis when large numbers of samples are needed to be analyzed to get precise information (Fang and Ramasamy, 2015). Whereas, indirect methods estimate the plant diseases by measuring the morphological and physiological changes or compounds released by infected plants in their defense (Golhani et al., 2018). The most popular indirect methods such as ML approaches offer a wide range of techniques for the detection of plant diseases (Golhani et al., 2018). The advantages and disadvantages of different types of detection methods for charcoal rot disease in crops are listed in Supplementary Table 2.

In agriculture research, ML methods are mainly used to detect, identify, and predict crop diseases and plant stress phenotyping (Yang and Guo, 2017). An efficient and precise prediction of plant diseases is a prerequisite in plant protection management. Moreover, early detection of disease minimizes the interference of humans (Golhani et al., 2018) which has been recently employed successfully (Saleem et al., 2019). However, prediction and quantification of charcoal rot disease are more crucial than the identification and classification of this disease in the future due to the implications of precise agriculture (Nagasubramanian et al., 2018). Such research works could lead to prevent the crop diseases at an early stage and cut costs of the pesticides (Barbedo, 2018).

In this work, specialized ML models were developed, for identification of charcoal rot disease by scrutinizing the symptoms of different parts of the soybean plants. In consequence of the lack of dataset for this disease, we have created our dataset; details of the dataset are provided in the dataset section. The main advantage of our proposed method is the identification of soybean charcoal rot disease at its early stage. A database of 2,000 soybean plants in natural field conditions was established. Supervised ML classifiers of LR-L1 LR-L2 MLP, RF, GBT, and SVM were trained to differentiate the healthy and infected soybean plants. Among these models, GBT classifier achieved a success rate of 96.79% through the analysis of the suggested feature set.

The occurrence of charcoal rot disease is regular, and the type and the probability of the soybean disease change during the soybean growth. Therefore, different charcoal rot disease identification techniques can be established by using the developed methods in this study. Furthermore, the automated charcoal rot disease prediction can be realized by combining identification models and domain knowledge of soybean disease. It has been previously reported that image processing and computer vision techniques can help to identify plant diseases (Golhani et al., 2018). The accuracy of the classification along with the image pre-processing could yield 90.5% recognition rate (Azlah et al., 2019). Thus far, only a few studies have been carried out to predict the charcoal rot disease development onset (Nagasubramanian et al., 2018). An algorithm such as image classification and image segmentation are mostly used for diseased charcoal rot identification (Saleem et al., 2019). These algorithms are used to classify healthy and no healthy plant leaves and stems of soybean (Saleem et al., 2019). By using the SVM approach, the highest classification accuracy was 95.76% and F1-score was 87% to identify the charcoal rot disease in soybeans (Nagasubramanian et al., 2018).

Although image processing and ML have provided significant evidence in the early prediction of disease, but different illumination conditions impact their performance (Mujika et al., 2018). Therefore, physiological evaluations can help to tackle this challenge (Khanna et al., 2019). Presented results have shown the applicability of the physiological features for the prediction of charcoal rot in soybean. As stated in Table 2, the result after using hybrid features, compared with only morphological features detailed in Table 3 has slightly higher performance. Moreover, Table 4 indicates the feature ranking based on various ML models highlighted the importance of physiological features in disease prediction.

In terms of classification performance, all methods performed well. GBT was the best preforming classifier as it tries to sequentially improves the performance and also it includes the feature interactions in the learning. MLP had the lowest performance among others. It could be due to our small data size as neural networks usually needs larger data size to perform well. We note that the small size of the dataset and considering all features to have the same importance are the limitations of this study.

## Conclusion

This paper investigated different ML algorithms for soybean charcoal rot disease detection and classification using morphological, physiological features. In this research effort, we presented an evaluation and comparison of six ML techniques on predicating charcoal rot disease. The results indicated that various ML techniques were slightly different in terms of their performance considering different evaluation metrics. Quantitative analysis of results indicated that GBT and SVM performed almost the same and demonstrated better performance compared with LR-L1, LR-L2, MLP, and RF approaches. Moreover, the feature ranking has shown the importance of including various features in the learning. Including other feature types such as chemical compositions and molecular structures and more data in the learning can be investigated as future work.

## Data Availability Statement

The datasets presented in this study can be found in online repositories. The names of the repository/repositories and accession number(s) can be found below: https://github.com/Elham-khalili/Soybean-Charcoal-Rot-Disease-Prediction-Dataset-code.

## Author Contributions

EK, SK, SR, and FG formulated the research problem and designed the approaches. EK performed the experiments and collected the dataset. EK wrote the initial draft of the paper. All authors contributed to the final draft of the paper and approved the final version of the manuscript. EK, SK, and SR developed the processing workflow and performed the data analytics. All authors contributed to the writing and development of the manuscript. All authors contributed to the article and approved the submitted version.

## Conflict of Interest

The authors declare that the research was conducted in the absence of any commercial or financial relationships that could be construed as a potential conflict of interest.

## Abbreviations

AUC: Area under the ROC curve
ELISA: Enzyme-linked immunosorbent assay
FCM: Flow cytometry
FISH: Fluorescence *in situ* hybridization
FN: False negatives
FP: False positives
GBT: Gradient tree boosting
IF: Immunofluorescence
LR: Regularized logistic regression
MCC: Matthews correlation coefficient
ML: Machine learning
MLP: Multilayer perceptron
PPV: Precision or positive prediction value
R7: Yellowing of the leaves and yellow pods at 50% growing stage
RF: Random forest
ROC: Receiver operating characteristic
RT-PCR: Polymerase chain reaction
SVM: Support vector machines
TN: True negatives
TP: True positive
t-SNE: t-distributed stochastic neighbor embedding
WB: Western blotting.
