# Supplementary figures and images for: Machine Learning Techniques for Soybean Charcoal Rot Disease Prediction
Source: Front Plant Sci. 2020 Dec 14;11:590529. doi: 10.3389/fpls.2020.590529 (PMC7767839; doi:10.3389/fpls.2020.590529)

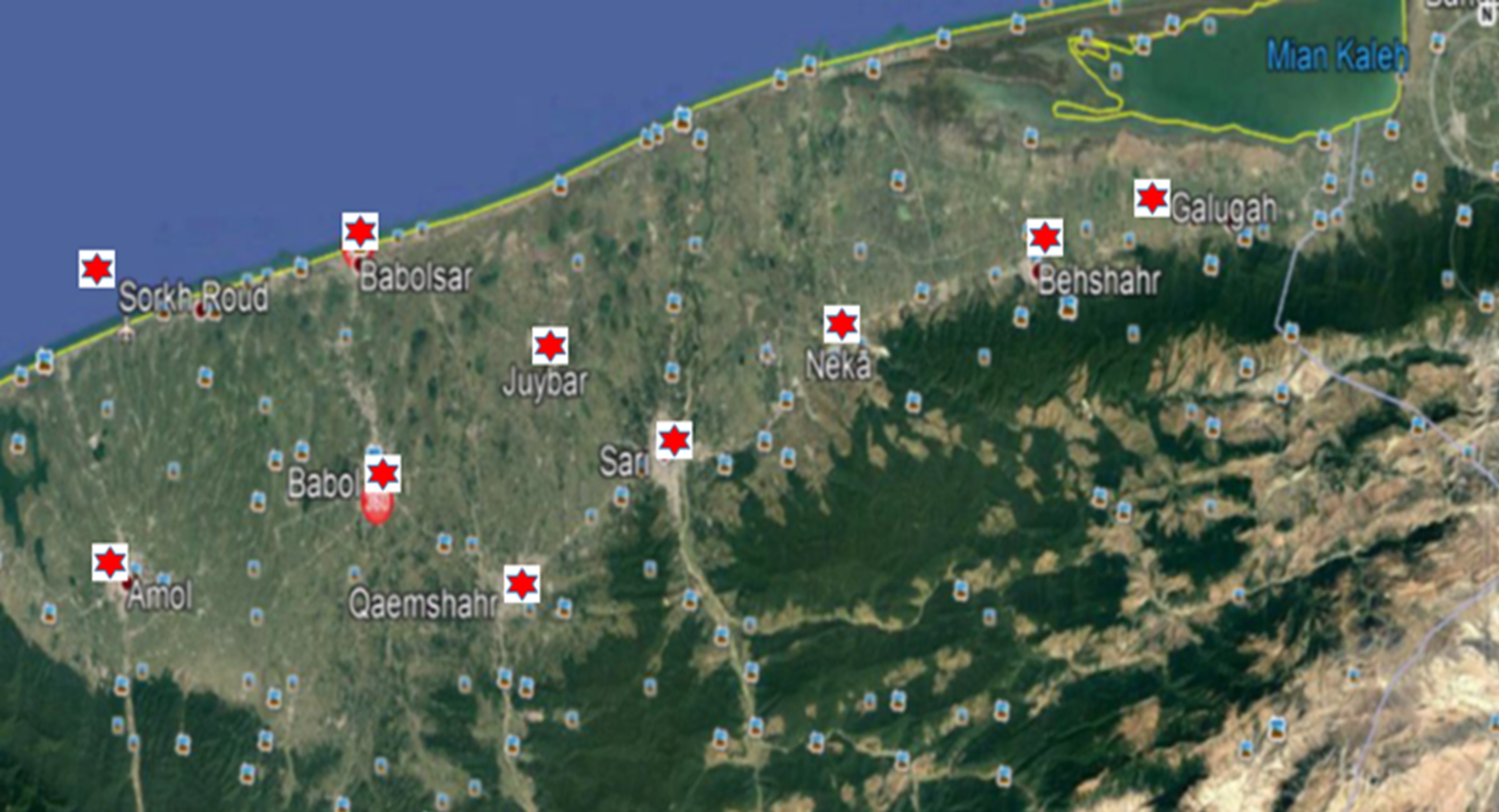

Supplement: Supplementary file 1 [file Image_1.JPEG]

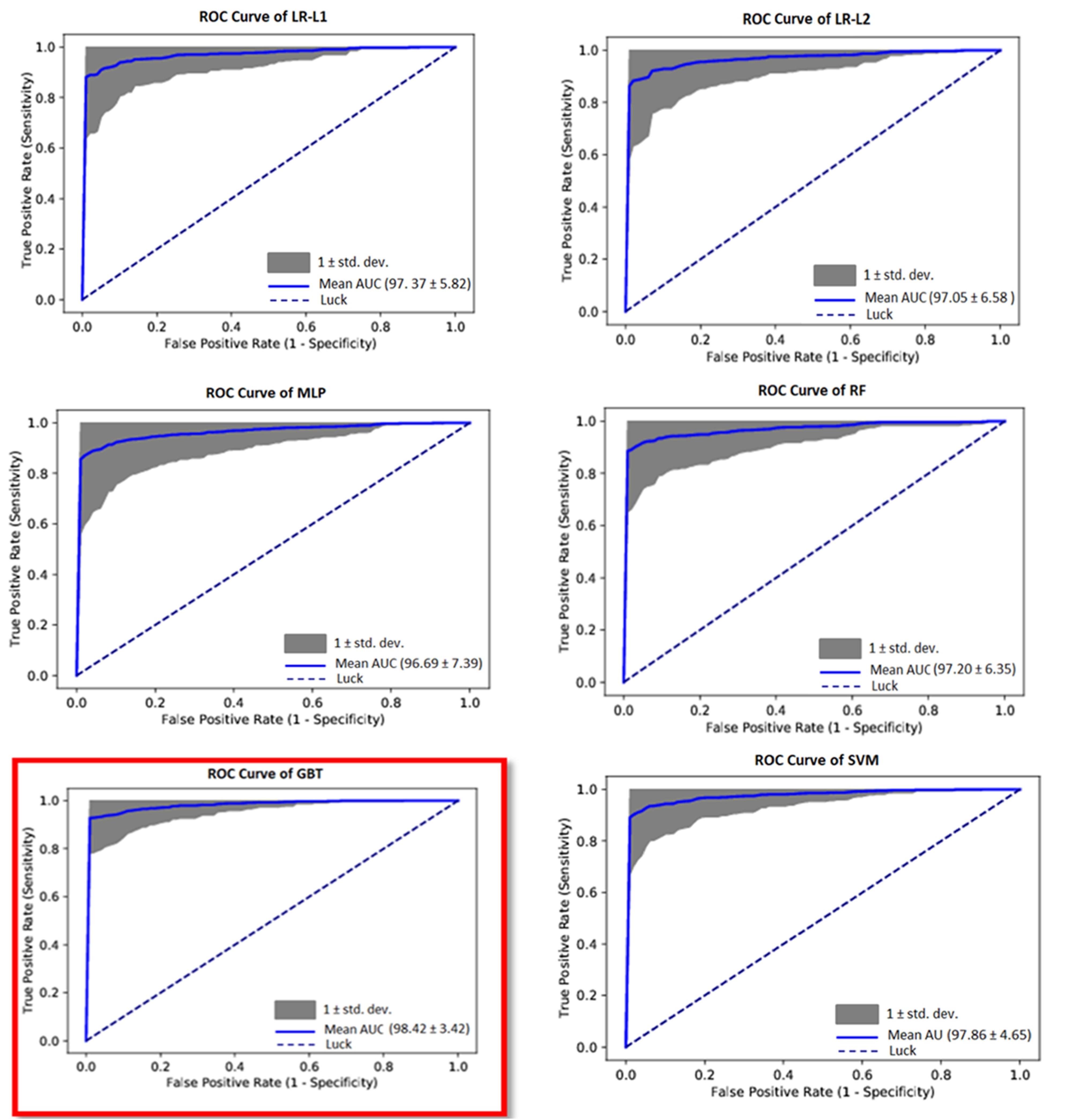

Supplement: Supplementary file 2 [file Image_2.JPEG]
