# Supplementary material for: Machine Learning Techniques for Soybean Charcoal Rot Disease Prediction
Source: Front Plant Sci. 2020 Dec 14;11:590529. doi: 10.3389/fpls.2020.590529 (PMC7767839; doi:10.3389/fpls.2020.590529)
Supplement: Supplementary file 3 [file Data_Sheet_1.PDF]

Supplementary Material

Supplementary Table 1. The dataset considered for the prediction of soybean charcoal rot disease.

Location	Number of healthy plants used for validation	Number of infected plants used for validation
Juybar	177	30
Neka	147	53
Amol	143	84
Babol	121	99
Sorkh Roud	120	108
Kiakola	109	119
Sari	87	121
Behshahr	53	122
Qaemshahr	21	124
Babolsar	22	140
	1000	1000

Supplementary Table 2. Summary of the advantages and disadvantages of the current techniques in detecting charcoal rot disease.

Techniques	Advantages	Disadvantages
Polymerase Chain Reaction	• An automated system<br>• A convenient technique<br>• Portable<br>• Easy to operate	• Laborious and expensive<br>• Chances of contamination<br>• Interfering of primers and probes may reduce sensitivity<br>• Simultaneous thermal cycling and fluorescence detection may be expensive and complex<br>• Critical to the samples
Immunofluorescence	• Accurate<br>• Can be visualized<br>• Simple	• Photo bleaching<br>• Expensive equipment<br>• Lack of quantitation
Fluorescence In-situ Hybridization	• Utilization of the short supply tissue<br>• Routine usage for non-dividing cells<br>• High sensitivity	• DNA and RNA copies may cause a problem in the identification of targets<br>• To identify the particular sequences of DNA, the tailing of the porob is important which may cause an issue.<br>• Auto-fluorescence<br>• Photo bleaching
Flow Cytometry	• Numerous parameters can be measured at the same time.<br>• Efficient<br>• Overwhelming unnecessary information	• Expensive<br>• Time-consuming fixation process<br>• Low sensitivity and detection<br>• High material requirements
Enzyme-linked Immunosorbent Assay	• Easy to use<br>• Reproducible<br>• Accurate and efficient<br>• Price-effective test<br>• Innocuous and ecologically effective	• Low sensitivity for fungus<br>• Laborious and expensive when an antibody is prepared.<br>• An advanced technique but may get expensive when culture media are obligatory<br>• Sometimes an error in results due to complex procedure<br>• Inadequate obstructive of immobilized antigen may give incorrect results<br>• An instability of antibody may cause an error if proper precautions are not adopted.<br>• Difficult movability due to instability of antibody which needs refrigeration<br>• Destructive to the samples
Western Blotting	• Flexibility<br>• Adjustable transfer conditions<br>• The transfer of a broad range of molecular weight is supported at one time<br>• This technique is companionable with prolonged transfer times for proteins of large molecular weight	• Background can result from cross-reactivity of antibodies<br>• Gel preparation takes more time<br>• Detection is possible if a large amount of protein is available<br>• Large volumes of transfer buffer are required<br>• When buffer gets hot, it affects the transfer, thus cooling (Machine or room) is required during transfer
Machine Learning	• Efficient and easy to operate.<br>• Prediction accuracy is high<br>• Disease-specific<br>• A limited hazard of harm to non-target plant<br>• Less expensive than other techniques<br>• Minimum safety measures required<br>• Repetition is not required due to its efficiency and accuracy.<br>• Durable solution<br>• It is powerful and can be adapted to complex functions<br>• It is adaptable to any situation	• Require large training data<br>• Requires data curation<br>• Possibility of overfitting to the observed data

Supplementary Figure 1. Soybean growing locations in Mazandaran Province, Iran; stars shows collection locations of healthy and infected soybean plants

Supplementary Figure 2. ROC curves of different ML approaches; AUC for each feature is given in the parentheses.

.
